# Multivalvular Cardiac Involvement from Giant Hepatic Metastases of an Ileal Neuroendocrine Tumor

**DOI:** 10.7759/cureus.103210

**Published:** 2026-02-08

**Authors:** Mario J Recio Ibarz, David Gómez Martín, Marta Matamala Adell, Andrea Carilla Sanromán, Luis M Álvarez De La Fuente

**Affiliations:** 1 Cardiology, Hospital Universitario Miguel Servet, Zaragoza, ESP; 2 Cardiac Surgery, Hospital Universitario Miguel Servet, Zaragoza, ESP; 3 Pathology, Hospital Universitario Miguel Servet, Zaragoza, ESP

**Keywords:** carcinoid heart disease, carcinoid syndrome, cytoreductive surgery, heart valve diseases, hepatic metastasis, intestinal neoplasms, multidisciplinary approach, neuroendocrine tumors, peptide receptor radionuclide therapy, somatostatin analogs

## Abstract

Intestinal neuroendocrine tumors (NETs) are rare, slow-growing neoplasms arising from enterochromaffin cells, capable of secreting vasoactive substances that may cause carcinoid syndrome (CS) and clinically significant valvular heart disease. These tumors are often diagnosed at advanced stages due to nonspecific gastrointestinal symptoms, and distant metastases, particularly to the liver and lymph nodes, are common at presentation. Hormonal dysregulation can lead to chronic diarrhea, flushing, and bronchospasm, while carcinoid heart disease (CHD) contributes substantially to morbidity and mortality, typically affecting right-sided valves, with left-sided involvement being uncommon and more frequently associated with intracardiac shunts than exceptionally high serotonin exposure.

Recent advances in management emphasize a multidisciplinary approach integrating systemic therapy, surgery, and targeted radionuclide treatment. Somatostatin analog therapy remains the cornerstone for controlling hormonal symptoms and slowing tumor progression. Aggressive cytoreductive surgery to achieve hormonal stabilization prior to surgical valve replacement has been associated with improved survival, symptomatic relief, and cardiac function. Targeted peptide receptor radionuclide therapy provides additional treatment for residual or metastatic disease, enhancing biochemical and radiological control.

We report the case of a 61-year-old woman with chronic diarrhea, weight loss, and recurrent flushing, diagnosed with a well-differentiated ileal NET with extensive hepatic metastases and severe right-sided valvular disease with mild left-sided involvement. She underwent somatostatin analog therapy to control hormonal symptoms, cytoreductive surgery, and surgical replacement of the pulmonary and tricuspid valves. Subsequent targeted peptide receptor radionuclide therapy further reduced tumor burden and stabilized biochemical markers. This combined multimodal strategy resulted in sustained clinical improvement, normalization of neuroendocrine markers, and long-term oncologic stability, as the patient remains asymptomatic and oncologically stable after four years of follow-up from initial presentation.

This case highlights the importance of early recognition, comprehensive evaluation, and a multidisciplinary strategy in advanced NETs. Coordinated care integrating endocrinology, oncology, cardiology, nuclear medicine, and surgery can significantly improve survival, hormonal control, and quality of life in patients with complex metastatic disease.

## Introduction

Gastrointestinal neuroendocrine tumors (NETs) are rare neoplasms arising from the diffuse neuroendocrine system, most frequently originating in the ileum from serotonin-producing enterochromaffin cells [1-3]. Due to their indolent growth and nonspecific early symptoms, these typically well-differentiated tumors are often diagnosed at advanced stages with lymph nodes and liver metastases [3,4].

Hepatic dissemination allows vasoactive substances to bypass hepatic metabolism, leading to carcinoid syndrome (CS) with flushing, diarrhea, and bronchospasm, and may progress to carcinoid heart disease (CHD) in approximately 20% to 50% of patients with established CS, representing a major determinant of morbidity and prognosis. CHD is characterized by predominantly right-sided valvular fibrosis and dysfunction. Left-sided valvular involvement is rare, due to pulmonary inactivation of circulating serotonin, and is more frequently associated with intracardiac shunts or, less commonly, with exceptionally high serotonin exposure [4].

Accurate staging and follow-up rely on integrating anatomical imaging with somatostatin receptor-based functional techniques, while echocardiography is essential for detecting and monitoring cardiac involvement [5-7]. Recent advances support a multidisciplinary approach combining somatostatin analog therapy, cytoreductive surgery to reduce tumor burden, and peptide receptor radionuclide therapy, resulting in improved hormonal control, progression-free survival, and quality of life in patients with advanced disease [8-11]. However, reports addressing advanced CHD with multivalvular involvement remain limited, particularly with respect to the timing, sequencing, and rationale of combined oncologic and cardiac surgical interventions [12,13].

We report the case of a 61-year-old woman with a well-differentiated ileal NET, extensive metastases, severe CS, and advanced right-sided CHD with mild left-sided involvement, successfully managed through a multimodal, multidisciplinary therapeutic strategy, thus providing insight into surgical decision-making and outcomes in advanced CHD.

## Case presentation

A woman with a history of long-standing iron deficiency presented with an 18-month history of chronic diarrhea, reporting up to 10 watery bowel movements per day, accompanied by a 20 kg weight loss. She also described generalized asthenia, an irritative cough, and progressively worsening exertional dyspnea. On physical examination, a systolic-diastolic murmur was noted along the left sternal border, as well as abdominal distension with a striking left hepatic lobe enlargement and bilateral lower limb edema.

**Figure 1:**
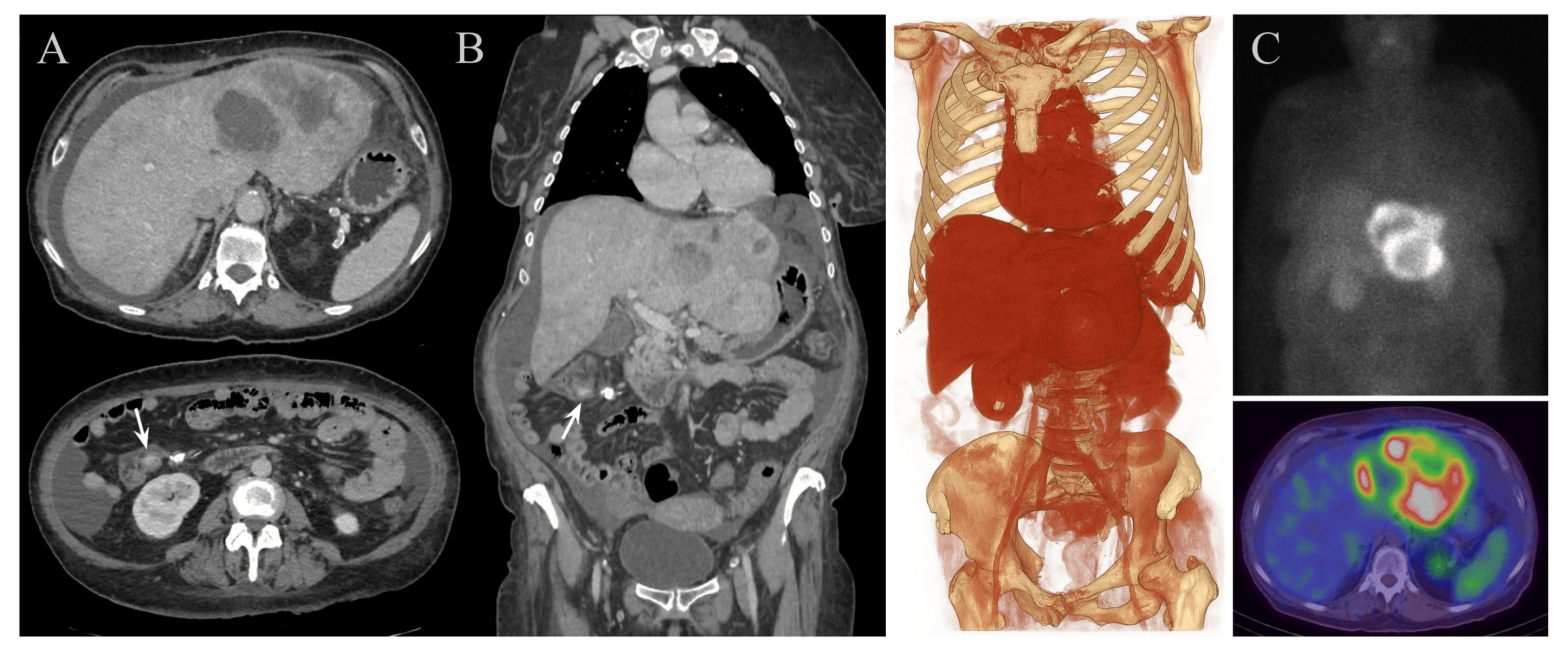
Computed tomography (CT) and single-photon emission computed tomography (SPECT) images of the metastatic tumor. (A-B) Contrast-enhanced CT images in the portal phase shown in axial and coronal planes, respectively, and a 3D volumetric reconstruction demonstrating two large metastases with necrotic centers in the left hepatic lobe, measuring 88 and 94 mm, respectively; mild ascites; and a hyperenhancing nodular lesion (arrow) of approximately 12 mm arising from the terminal ileum near the ileocecal valve, with an adjacent calcified lymphadenopathy and a circumferential mesenteric desmoplastic reaction (“wheel-spoke” pattern), highly suggestive of a metastatic intestinal carcinoid tumor. (C) SPECT with technetium-99m-hydrazinonicotinyl-Tyr³-octreotide (⁹⁹mTc-HYNIC-TOC) demonstrating somatostatin receptor expression at the margins of the hepatic metastases, visible both in volumetric reconstructions and on fused CT axial images.

Upper and lower gastrointestinal endoscopies with biopsies were performed, with no significant findings (performed at an outside institution; images not available for review). Following the administration of intravenous sedation, the patient developed facial and truncal flushing, which she reported as a recurrent symptom for several years. A computed tomography (CT) scan was then performed to evaluate a suspected CS, revealing two large hyperproliferative lesions in the left hepatic lobe, along with mild ascites, omental stranding, and calcified ileocecal lymphadenopathy adjacent to a small hyperenhancing focus in the terminal ileum (Figure 1).

**Figure 2:**
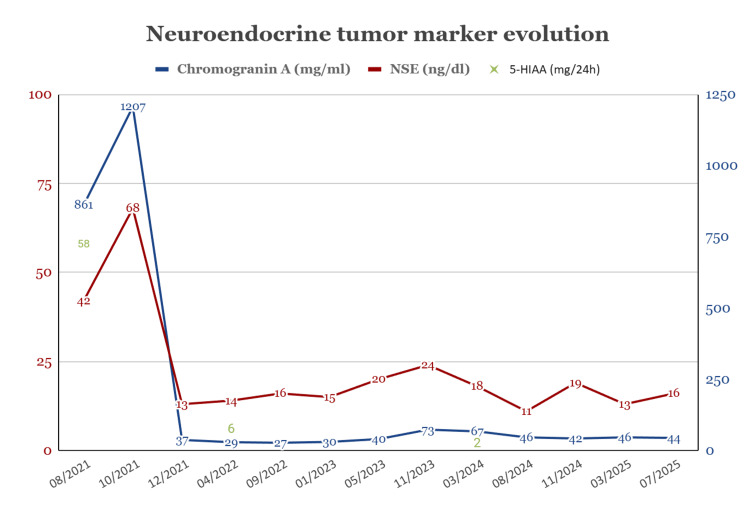
Neuroendocrine tumor marker evolution Line graph illustrating temporal evolution of serum chromogranin A and neuron-specific enolase (NSE; normal values of ≤ 98 md/dL and 12 ng/dL, respectively) and 24-hour urine 5-hydroxyindoleacetic acid (normal value of < 8 mg/24 hours), depicting their reduction after cytoreductive oncologic surgery (11/2021), as well as the progressive mild increase, reduction and subsequent stability after peptide receptor radionuclide therapy (12/2023–06/2024), up to the time of manuscript preparation.

Serum chromogranin A and neuron-specific enolase (NSE) levels were elevated (at 1207 mg/mL and 68 ng/dL, with normal values of ≤ 98 mg/mL and 12 ng/dL, respectively), as well as urinary 5-hydroxyindoleacetic acid (5-HIAA at 58 mg/24 hours, with normal value of < 8 mg/24 hours) (Figure 2).

A percutaneous liver biopsy was consistent with a well-differentiated intestinal NET with a low proliferative index. Other endocrine tumors were excluded, and treatment with the somatostatin analogues (SSA) lanreotide and octreotide was initiated, resulting in partial clinical improvement.

Single-photon emission computed tomography (SPECT) with technetium-99m-hydrazinonicotinyl-Tyr³-octreotide (⁹⁹mTc-HYNIC-TOC) was performed, indicating somatostatin receptor expression in the hepatic lesions (Figure 1). A positron emission tomography (PET) scan with ^68Ga-DOTA-peptides demonstrated uptake in the hepatic lesions, terminal ileum, and a pulmonary nodule, with maximum standardized uptake values (SUVmax) of 23, 3.1, and 1.5, respectively (performed at an outside institution; images not available for review).

**Figure 3:**
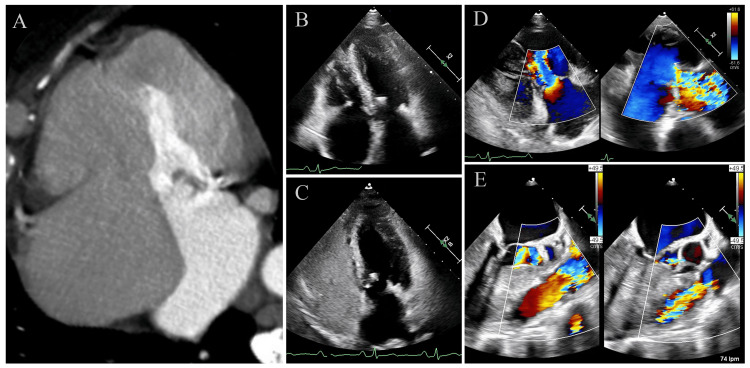
Carcinoid-related valvular heart disease (A) Axial, arterial-phase contrast-enhanced computed tomography images, showing marked dilation of the right-sided heart chambers with flattening of the interventricular septum and leftward displacement of the interatrial septum secondary to right-sided strain, as well as calcification of the mitral valve and annulus. (B–C) Transthoracic echocardiography images in the apical four-chamber view during early diastole, showing right chamber dilation and mitral-tricuspid valvular degeneration, with extensive carcinoid-related calcification of both valves and the right subvalvular apparatus, as well as no bubble passage to the left chambers. (D–E) Transesophageal echocardiography images with color Doppler in the mid-esophageal view of the tricuspid valve (105°) and pulmonary valve (120°), both demonstrating mild opening restriction and severe regurgitation.

The patient was referred to the cardio-oncology clinic for evaluation. Transthoracic echocardiography (TTE), followed by transesophageal echocardiography, revealed severe organic pulmonary and tricuspid valvular impairment, with both valves exhibiting mild stenosis and severe regurgitation, along with mild involvement of the mitral and aortic valves. The right ventricle was mildly dilated with preserved systolic function, while the left ventricle was mildly hypertrophic, with preserved dimensions and systolic function. Indirect findings were suggestive of moderate pulmonary arterial hypertension, and the bubble test was negative (Figure 3, Appendix A). Diuretic therapy with furosemide and neurohormonal therapy with losartan, dapagliflozin, and spironolactone were initiated, leading to relative improvement in congestive symptoms.

**Figure 4:**
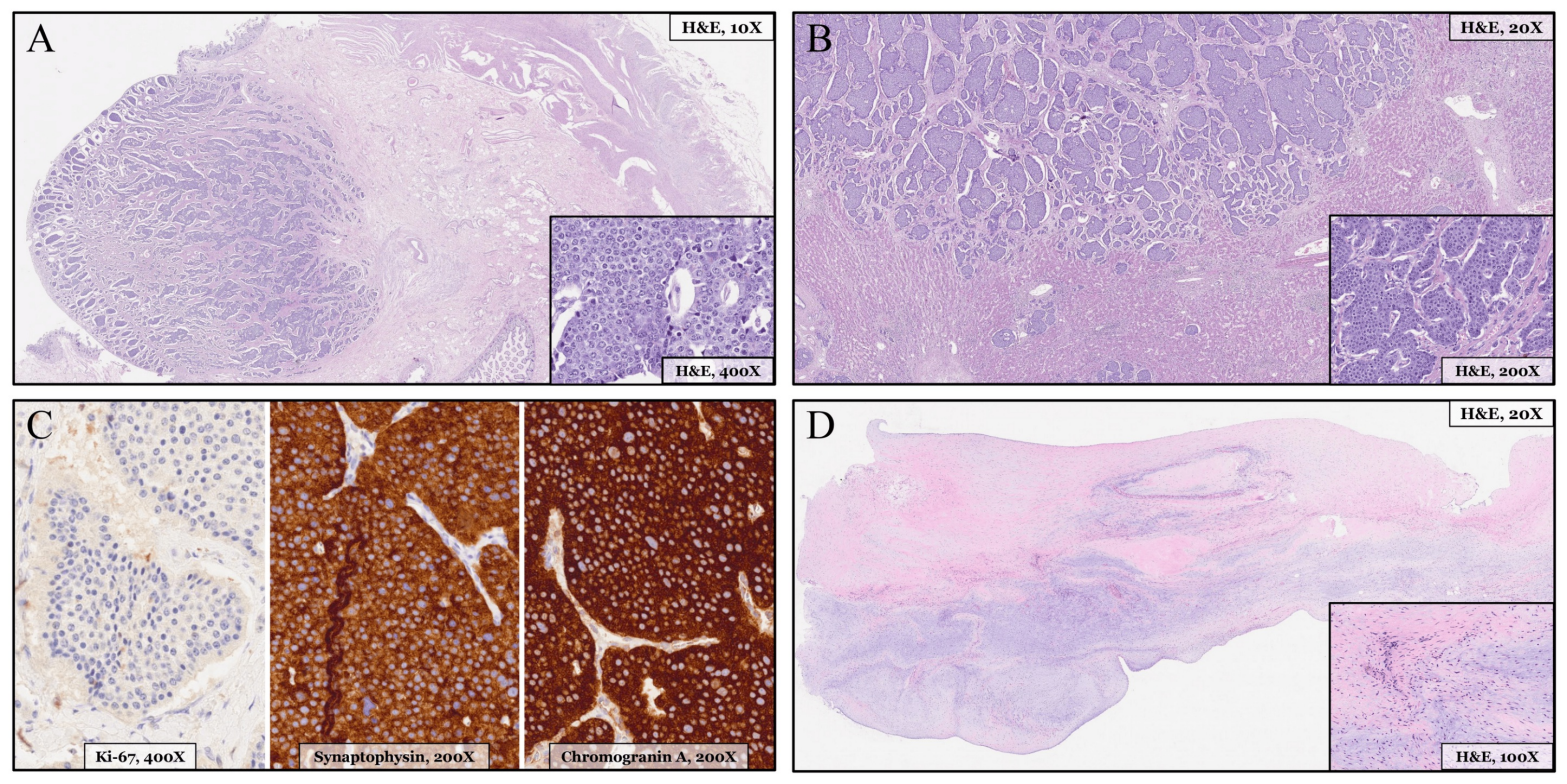
Histopathological analysis of tumor and valvular tissue (A) Hematoxylin-eosin (H&E) histopathological staining of an ileum section adjacent to the ileocecal valve, with a well-circumscribed solid nodular tumor lesion (left part) infiltrating the intestinal mucosa and submucosa, composed of solid nests separated by fibrous septa, with detail of the small, uniform, round tumor cells with eosinophilic cytoplasm and small round nuclei, exhibiting a characteristic “salt-and-pepper” chromatin pattern. (B) H&E staining of a hepatic section, with extensive tumor infiltration (upper part) composed of solid nests with cytological features consistent with metastatic intestinal neuroendocrine tumor, contrasted with the residual healthy hepatic parenchyma (lower part). (C) Immunohistochemical staining of tumor metastases, demonstrating proliferative index assessment by Ki-67 (<3%) and cytoplasmic positivity for chromogranin A and synaptophysin. (D) H&E staining of a tricuspid leaflet section, with thickening secondary to fibromyxoid-type degenerative changes, and detail of the basophilic stroma.

A multidisciplinary tumor board decided on digestive surgical intervention, including segmental ileal resection, right hemicolectomy, D3 lymphadenectomy, left hepatectomy, partial segment 8 resection, and cholecystectomy. Postoperative recovery was prolonged but without major complications. Histopathological examination revealed a well-differentiated 8 mm intestinal NET originating from the ileal submucosa (Figures 4A-4C), with perineural, lymphatic, and submucosal infiltration, various peritoneal implants, and the previously known multifocal hepatic metastases (Figure 4B). No evident nodal involvement was observed (stage IV, pT1aN0M1). The patient continued treatment with lanreotide, remaining asymptomatic and oncologically stable, with near normalization of tumor markers (Figure 2) over 1.5 years of follow-up.

After achieving oncologic control, coronary artery disease was excluded by coronary angiography, and right heart catheterization was performed as part of preoperative planning. Findings included a preserved mean right atrial pressure (at 6 mmHg with a V wave reaching 12 mmHg, thus reflecting a significant tricuspid regurgitation); a right ventricle with moderately elevated systolic and normal diastolic pressure (at 47/5 mmHg, consistent with an adequate adaptation to volume overload); normal pulmonary artery pressures (at 23/14/8 mmHg) and capillary wedge pressure (at 9 mmHg); preserved left-sided pressures and cardiac output; and no evidence of an intracardiac shunt.

**Figure 5:**
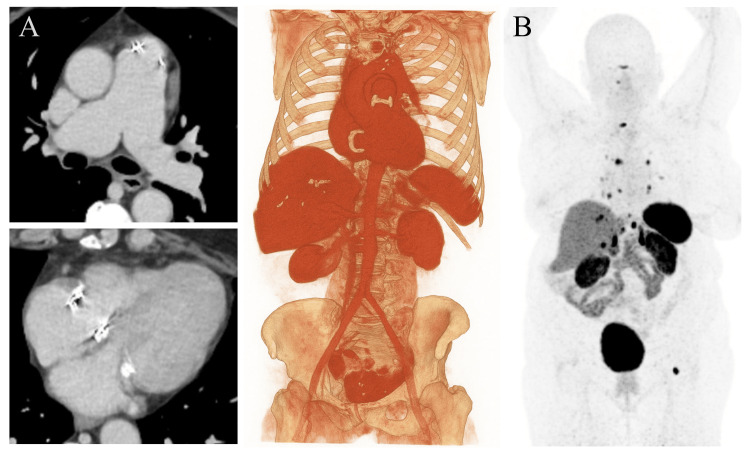
Imaging assessment of valvular replacement and cytoreductive oncologic surgery outcomes. (A) Contrast-enhanced computed tomography in the portal phase and axial plane, respectively depicting the tricuspid and pulmonary valve bioprostheses with reverse remodeling of the right heart chambers and a residual pulmonary artery aneurysm, together with a 3D volumetric reconstruction illustrating the result of lymphadenectomy, cholecystectomy, intestinal resection, and hepatectomy. (B) PET with ^68Ga-DOTA-peptides demonstrating foci of increased uptake (Krenning score 3) in the liver, lungs, bones, and lymph nodes, suggestive of disease spread, almost two years after tumor cytoreductive surgery.

A multidisciplinary team decided on surgical replacement of the pulmonary and tricuspid valves with bioprostheses (Figure 5). Although there appeared to be some carcinoid-related involvement of the mitral and aortic valves, these valves were left intact because of minimal functional impact and the already elevated baseline surgical risk. The postoperative course was uneventful, and histopathological sample examination of the excised valves demonstrated fibromyxoid degenerative changes (Figure 4D), without fully developed carcinoid-specific plaques.

In the context of progressively rising tumor markers (Figure 2) and the onset of cough and facial flushing, a repeat PET-CT scan (Figure 5) with ⁶⁸Ga-DOTA-peptides confirmed oncological progression, showing foci of increased uptake (Krenning score 3) in the liver, lungs, bones, and lymph nodes. The patient subsequently underwent four cycles of radionuclide therapy with ^177Lu-DOTATATE, alternating with lanreotide, which was well tolerated. Two years after valve replacement and radionuclide therapy, at the time of manuscript preparation, the patient shows no evidence of tumor progression, has adequate control of serotonergic symptoms, maintains excellent functional status and quality of life, and is under a regular six-month follow-up in a dedicated cardio-oncology clinic.

## Discussion

Gastrointestinal tract NETs are considered rare malignancies, with a reported annual incidence of approximately three to five cases per 100,000 inhabitants, whereas their prevalence is substantially higher due to prolonged survival associated with their typically indolent course, exceeding 30 per 100,000 in contemporary series [1]. They are most frequently located in the appendix and small intestine, particularly the ileum [2], and derive from enterochromaffin cells predominantly situated in the submucosa. These components of the diffuse neuroendocrine system contain secretory granules that release serotonin and other bioactive mediators responsible for the typical clinical manifestations of CS, including flushing, diarrhea, and bronchospasm [3].

Ileal NETs, usually well differentiated, are characterized by slow growth and an indolent clinical course until advanced stages [4]. A significant proportion of patients present with metastases at diagnosis, most frequently to the liver, lymph nodes, bone, lung, and peritoneum [3]. The presence of hepatic metastases is particularly relevant, as vasoactive mediators can bypass hepatic metabolism, thereby promoting the development of CS and CHD. In this context, reported estimates largely reflect CHD prevalence rather than true incidence, as it develops over time in patients with sustained serotonergic exposure, affecting approximately 20% to 50% of patients with established CS [4].

From a pathophysiological perspective, these substances stimulate fibroblasts and myofibroblasts, promoting extracellular matrix deposition [2]. At the mesenteric level, this peritumoral desmoplastic reaction can lead to fibrotic mesenteric retraction and local ischemic complications, typically recognized on imaging as a spiculated “wheel-spoke” pattern. In our patient, despite the presence of calcified ileocecal lymphadenopathy on preoperative imaging, no viable nodal metastases were identified on histopathological examination. This imaging-pathology discordance highlights the potential for false-negative histopathological findings, as nodal calcification and desmoplasia may persist despite the absence of viable tumor cells or sampling-related underrepresentation [5]. Endocardial fibrosis promotes retraction of cardiac valve leaflets and chordae with a predilection for the right-sided structures, due to pulmonary metabolism of circulating tumor products [1]. Left-sided involvement is rare and classically associated with intracardiac shunting, although it may also occur with prolonged exposure to exceptionally high circulating serotonin levels, possibly due to saturation of pulmonary monoamine oxidase. This pattern may thus indicate advanced disease burden and warrants careful surveillance, as progression of left-sided involvement may influence treatment decisions and prognosis [4].

For imaging diagnosis, although CT is the initial technique, optimal staging of these patients combines molecular studies targeting somatostatin receptors. PET/CT with ⁶⁸Ga-DOTA-peptides has demonstrated higher sensitivity than SPECT/CT with ⁹⁹mTc-HYNIC-TOC, thanks to its superior spatial resolution, and is considered the technique of choice to characterize the extent and recurrence of well-differentiated NETs [6]. In the cardiology setting, echocardiography (particularly TTE) is essential due to its accessibility, noninvasive nature, and ability to assess and longitudinally monitor valvular involvement, pulmonary pressure, ventricular function, and remodeling, as well as to rule out intra- and extracardiac shunting [7].

The prognosis of metastatic NETs is variable and depends mainly on factors such as cardiac involvement, tumor burden, histological differentiation, and hormonal control [4]. Aggressive cytoreductive surgery, particularly when complete tumor resection is achieved, has recently been associated with improved overall survival and symptomatic control of CS. Thus, the presence of peritoneal metastases should not be considered an absolute contraindication to surgical intervention [8]. SSAs constitute a first-line pharmacological treatment for well-differentiated NETs, especially of intestinal origin. In addition to their symptomatic effect, they possess antiproliferative properties that can delay tumor progression [9,10]. Radionuclide therapy with ¹⁷⁷Lu-DOTATATE has demonstrated significant benefits in progression-free survival, radiological response rate, and quality of life in patients with disease progression despite SSA therapy [11]. In this case, it was initiated in the setting of documented clinical, imaging, and biochemical progression (as described in Figure 2), once postoperative cardiac status had stabilized.

The treatment of choice for CHD with severe valvular involvement is surgical replacement, combined with closure of any intracardiac shunting, and always supported by baseline neurohormonal therapy [4,12]. Current expert recommendations support achieving hormonal and oncologic control before cardiac surgery to reduce perioperative risk [13]. Bioprosthetic valves are preferred, as they carry a lower risk of thrombosis and eliminate the need for long-term anticoagulation in patients with hepatic dysfunction, although they are associated with a higher-than-usual risk of prosthetic degeneration, particularly in the setting of suboptimal prior oncologic or hormonal control. Although pulmonary valve replacement is less standardized than tricuspid valve replacement in CHD, in the present case, it was undertaken to prevent persistent right ventricular volume overload in the setting of severe pulmonary regurgitation, a strategy supported by emerging surgical series in advanced CHD [12,13].

Therefore, the management of patients with CS and CHD requires a multidisciplinary approach involving, among others, specialists in oncology, digestive surgery, cardiology, cardiac surgery, nuclear medicine, radiology, and pathology. This approach enables integration of tumor and biochemical control, optimization of cardiac function, and careful timing of surgical intervention, factors shown to improve both patient survival and quality of life [13].

## Conclusions

Metastatic intestinal NETs require a comprehensive, multidisciplinary approach combining pharmacological and surgical treatments. Cardiological assessment, together with valve replacement surgery when indicated, has a significant prognostic impact in CHD, particularly in cases with multivalvular involvement, including uncommon left-sided disease. The use of both anatomical and functional imaging techniques, along with histological and biochemical evaluation, allows for individualized therapy and precise prognostic stratification. In this context, the present case supports the feasibility and benefit of combined surgical and radionuclide management and illustrates how appropriate sequencing of oncologic control and cardiac intervention can achieve effective clinical, hormonal, and oncologic control. These findings may inform surgical decision-making and emphasize the importance of structured long-term cardiac and oncologic surveillance in similar high-risk patients, ultimately contributing to improved survival and quality of life in advanced disease with cardiovascular involvement associated with CS.

## Appendices

Appendix A 
